# Noncoding RNAs in gastric cancer: implications for drug resistance

**DOI:** 10.1186/s12943-020-01185-7

**Published:** 2020-03-19

**Authors:** Ling Wei, Jujie Sun, Nasha Zhang, Yan Zheng, Xingwu Wang, Liyan Lv, Jiandong Liu, Yeyang Xu, Yue Shen, Ming Yang

**Affiliations:** 1 Shandong Provincial Key Laboratory of Radiation Oncology, Cancer Research Center, Shandong Cancer Hospital and Institute, Shandong First Medical University and Shandong Academy of Medical Sciences, Jinan, 250117 Shandong Province China; 2 Department of Pathology, Shandong Cancer Hospital and Institute, Shandong First Medical University and Shandong Academy of Medical Sciences, Jinan, 250117 Shandong Province China; 3 Department of Radiation Oncology, Shandong Cancer Hospital and Institute, Shandong First Medical University and Shandong Academy of Medical Sciences, Jinan, 250117 Shandong Province China

**Keywords:** Gastric cancer, Drug resistance, MicroRNA, Long non-coding RNA, Circular RNA

## Abstract

Gastric cancer is the fourth most common malignancy and the third leading cause of cancer-related deaths worldwide. Advanced gastric cancer patients can notably benefit from chemotherapy including adriamycin, platinum drugs, 5-fluorouracil, vincristine, and paclitaxel as well as targeted therapy drugs. Nevertheless, primary drug resistance or acquisition drug resistance eventually lead to treatment failure and poor outcomes of the gastric cancer patients. The detailed mechanisms involved in gastric cancer drug resistance have been revealed. Interestingly, different noncoding RNAs (ncRNAs), such as microRNAs (miRNAs), long noncoding RNAs (lncRNAs) and circular RNAs (circRNAs), are critically involved in gastric cancer development. Multiple lines of evidences demonstrated that ncRNAs play a vital role in gastric cancer resistance to chemotherapy reagents and targeted therapy drugs. In this review, we systematically summarized the emerging role and detailed molecular mechanisms of ncRNAs impact drug resistance of gastric cancer. Additionally, we propose the potential clinical implications of ncRNAs as novel therapeutic targets and prognostic biomarkers for gastric cancer.

## Background

Gastric cancer is a malignant tumor originating from the gastric mucosa. As the fourth most common malignancy and the third leading cause of cancer-related deaths worldwide, there were about 984,000 new gastric cancer cases and 841,000 deaths occurred in 2013 [1]. Due to lack of obvious and specific symptoms at early disease stage, most gastric cancer patients were diagnosed at advanced disease stages with poor prognosis. The combined use of chemotherapy and targeted therapy has evidently prolonged the overall survival and improved life quality of gastric cancer patients with advanced disease. The commonly used chemotherapeutic agents include adriamycin (ADR), platinum drugs, 5-fluorouracil (5-FU), vincristine (VCR) and paclitaxel (PTX). However, the development of multi-drug resistance (MDR) of gastric cancer cells is a major hurdle in clinical oncology, which may result in a poor prognosis. The complicated mechanisms involved in gastric cancer MDR include inactivation of apoptosis signaling pathways, loss of cell cycle checkpoint control, accelerated cell proliferation and autophagy flux, enhanced DNA damage repair capacity, diminished uptake and/or increased efflux of drugs via upregulated MDR-associated proteins, activated cancer stem cells (CSCs), as well as epithelial-mesenchymal transition (EMT) [2–4]. However, the detailed mechanisms of MDR remain inconclusive.

Noncoding RNAs (ncRNAs) are RNA transcripts encoding no proteins and are classified as small ncRNAs (sncRNAs, 18~200 nt) and long noncoding RNAs (lncRNAs, >200 nt). There are different kinds of sncRNAs, such as microRNAs (miRNAs), small nuclear RNAs (snRNAs), piwi interacting RNAs (piRNAs), and small nucleolar RNAs (snoRNAs) [5–15]. It has been found that ncRNAs are involved in almost all cellular functions, such as proliferation, apoptosis, EMT, autophagy and cell cycle control [16–20]. Multiple ncRNAs acts as oncogenes or tumor suppressor genes during carcinogenesis and can also serve as diagnostic and prognostic markers of cancer patients after certain therapies [21, 22]. In gastric cancer, a variety of abnormally expressed ncRNAs were identified to promote tumor progression, radioresistance, chemoresistance and targeted therapy sensitivity [23–28]. Interestingly, several miRNAs, lncRNAs and circRNAs have been found to play an essential part in gastric cancer drug resistance, especially in chemoresistance. However, there are still no studies how lncRNAs and circRNAs are involved in gastric cancer resistance to targeted therapy. Interestingly, it has been found that via sponging miR-331-3p in gastric cancer, lncRNA HOX transcript antisense gene RNA (HOTAIR) can regulate the expression of human epidermal growth factor receptor 2 (HER2), a target of targeted therapy agent [29]. Similarly, lncRNA H19 could regulate HER2 expression through sequestering let-7c in gastric cancer cells [30], indicating the potiential of ncRNAs may modulate sensitivity to targeted therapy.

Immunotherapy with immune checkpoint inhibitors for gastric cancer has emerged [31–37]. In gastric cancer, the relatively identified and useful population for immunotherapy is patients receiving third-line treatment. The commonly used immune-checkpoint blockade agents include anti-programmed cell death protein 1 (PD-1) monoclonal antibodies (Nivolumab and Pembrolizumab), anti-PD-L1 IgG1 antibody (Avelumab), as well as anti-CTLA-4 antibodies (Ipilimumab and Tremelimumab). Multiple parameters, such as microsatellite instability (MSI), mismatch repair (MMR) deficiency, high mutational load and high infiltrating CD8+ T cells density in tumor tissues can predict the response of solid tumors to immunotherapy [38–41]. To the best of our knowledge, there are no reports on miRNAs, lncRNAs and circRNAs involved in drug resistance to immune-checkpoint blockade agents in gastric cancer.

Herein, we performed a systematic literature review on the detailed mechanisms how ncRNAs intensify or weaken drug resistance of gastric cancer, which highlighting that ncRNAs may act as potential biomarkers and/or therapeutic targets of gastric cancer.

## MiRNAs and drug resistance

MiRNAs are a class of sncRNAs of 19-24 nt in length, which could post-transcriptionally suppress gene expression via binding to the 3'-untranslated region (3'-UTR) of multiple target messenger RNAs (mRNAs) and/or other RNAs [42, 43]. Multiple miRNAs are dysregulated in gastric cancer and play a crucial role in tumorigenesis, cancer metastasis, as well as development of resistance to drugs and radiation [44–48]. It has been observed that significantly changed miRNA expression profiles in drug-resistant gastric cancer cells compared to those in drug-sensitive cells. The involvement of miRNAs in gastric cancer resistance to ADR, platinum drugs, 5-FU, VCR, PTX and other drugs is summarized below.

### MiRNAs and ADR resistance

As a cell cycle non-specific anti-cancer antibiotic, ADR can inhibit the synthesis of nucleic acids through embedding into DNA and exert strong cytotoxic effects on tumor cells. In gastric cancer patients, ADR is commonly used in combination with platinum drugs, 5-FU, VCR, PTX and mitomycin. Multiple genes and miRNAs have been shown to be responsible for the development of ADR resistance in gastric cancer [49–53].

Several oncogenic miRNAs can promote ADR resistance in gastric cancer (Table 1). MiR-27a, for instance, has been found to confer ADR resistance by inhibiting multidrug resistance 1 (MDR1)/P-gp expression in gastric cancer cells [54]. Exogenous expression of miR-135a-5p could enhance apoptosis resistance to ADR in gastric cancer cells via silencing activating protein 2 alpha (AP-2α) and, thus, upregulating Bcl-2 expression [55]. Interestingly, Epstein-Barr (EB) virus miRNA miR-BART20-5p could also significantly increase ADR resistance of gastric cancer by inhibiting gene expression of BAD, a Bcl-2 family member [56]. MiR-19a/b, a member of the *miR-17-92* cluster, is highly expressed in SGC7901/ADR resistant gastric cancer cell. It has been found that miR-19a/b promotes ADR resistance of gastric cancer via targeting and suppressing expression phosphatase and tensin homologue (PTEN). Meanwhile, miR-19a/b could accelerate ADR efflux of gastric cancer cells by increasing P-gp expression and inhibit apoptosis induced by ADR [57]. MiR-501 is another significantly up-regulated miRNA in SGC7901/ADR cells. MiR-501 could confer ADR resistance of gastric cancer via suppressing expression of BH3-like motif-containing protein, cell death inducer (BLID) [58]. Additionally, miR-501 was also identified in SGC7901/ADR cells-secreted exosomes (ADR Exo) with a relatively high level. Exosome transferred miR-501 could promote tumorigenesis and ADR resistance through down-regulating BLID expression, and subsequently, inactivating phosphorylation of AKT and caspase-9/-3 [59]. MiR-20a was also proved to enhance gastric cancer resistance to ADR via inhibiting expression of early growth response 2 (EGR2), a member of a multi-gene family which encoding C2H2-type zinc-finger proteins [60]. Through silencing PTEN and tissue inhibitor of matrix metalloproteinases 3 (TIMP3), miR-21-5p increases ADR resistance of gastric cancer cells [61]. It has also been found that miR-633 promotes ADR resistance of gastric cancer by inhibiting expression of Fas-associated death domain (FADD) [62]. In addition, epigenetics controlling mediated by miRNAs has been reported as an important signaling impacting tumorigenesis of gastric cancer. The histone deacetylase (HDAC) family plays a key role in epigenetics regulation of gastric cancer [80]. For example, it has been found that miR-520h down-regulated expression of HDAC1, which contributing to ADR resistance of gastric cancer [63].

**Table 1:** MiRNAs and adriamycin resistance in gastric cancer

MiRNAs	Expression^1^	Genes and Pathways	References
miR-27a	↑	*P-gp*, *cyclin D1*, *p21*	[54]
miR-135a-5p	↑	*AP-2α*	[55]
miR-BART20-5p	↑	*Bad*	[56]
miR-19a/b	↑	*PTEN*	[57]
miR-501	↑	*BLID*	[58, 59]
miR-20a	↑	*EGR2*	[60]
miR-21-5p	↑	*PTEN,TIMP3*	[61]
miR-633	↑	*FADD*	[62]
miR-520h	↑	*HDAC1*	[63]
miR-129-5p	↓	*ABCB1*, *ABCC5*, *ABCG1*	[64]
miR-508-5p	↓	*ABCB1*, *ZNRD1*	[65]
miR-103/107	↓	*cav-1*, *P-gp*	[66]
miR-27b, miR-508-5p	↓	*ABCB1*, *CCNG1*, *ZNRD1*	[67]
miR-140	↓	*ABCC1*, *ABCG2*, *SOX4*	[68]
miR-107	↓	*P-gp*, *cyclin D1*, *c-myc*	[69]
miR-15b, miR-16	↓	*Bcl-2*	[70]
miR-181b	↓	*Bcl-2*	[71]
miR-497	↓	*Bcl-2*	[72]
miR-200bc/429	↓	*Bcl-2*, *XIAP*	[73]
miR-34	↓	*Bcl-2*, *Notch*, *HMGA2*	[74]
miR-200c	↓	*E-cadherin*, *Bax*, *Bcl-2*	[75]
miR-185	↓	*ARC*	[76]
miR-126	↓	*EZH2*	[77]
miR-218	↓	*SMO*	[78]
miR-494	↓	*PDE4D*	[79]

^1^miRNAs either up-regulated (↑) or down-regulated (↓) in adriamycin resistant gastric cancer cells

Note: This table shows 25 miRNAs whose expression levels and potential targets in adriamycin resistance of gastric cancer.

By contrast, multiple tumor suppressor miRNAs can reverse ADR resistance of gastric cancer (Table 1). For instance, ectopic miR-129-5p expression can weaken the ADR resistance in gastric cancer cells SGC7901/ADR via silencing ABCB1 (ATP-binding cassette, subfamily B, member 1), ABCC5 and ABCG1 [64]. Similarly, exogenous expression of miR-508-5p has been found to reverse ADR resistance and decrease the intracellular concentration of chemotherapeutics by inhibiting expression of ABCB1 and zinc ribbon domain-containing 1 (ZNRD1) [65]. Additionally, miR-103/107 has also been shown to inhibit P-gp function/expression and increase ADR sensitivity of SGC7901/ADR cells. Mechanically, caveolin-1 (cav-1), a key component of lipid rafts regulating P-gp activity, is considered as the direct target gene of miR-103/107 [66]. Tumor suppressor miR-27b, miR-140 and miR-107, have also been confirmed to be involved in ADR resistance of gastric cancer by modulating expression/function of the ABC transporters family members [67–69]. In addition to ABC transporters, the Bcl-2 family also participate in chemoresistance of malignancies, including gastric cancer. Tumor suppressors miR-15b, miR-16, miR-181b and miR-497 could promote ADR-induced apoptosis of gastric cancer cells via silencing Bcl-2 expression [70–72]. MiRNAs encoded by the *miR-200bc/429* cluster has also been found to function as inhibitors of ADR- resistant gastric cancer cells through inhibiting expression of Bcl-2 and X-linked inhibitor of apoptosis (XIAP) [73]. Restoration of miR-34 in gastric cancer cells suppresses growth and increased apoptosis through down-regulating expression of Bcl-2, Notch, and high-mobility group AT-hook 2 (HMGA2) [74]. Ectopic miR-200c could sensitize SGC7901/DDP cell to ADR, cisplatin (DDP) and 5-FU through suppressing expression of Bcl-2 and Bax expression [75]. Additionally, significant reduced expression of tumor suppressors miR-185, miR-126, miR-218 and miR-494 in ADR-resistant gastric cancer cells could diminish ADR sensitivity by silencing apoptosis repressor with caspase recruitment domain (ARC), enhancer of zeste homolog 2 (EZH2), smoothened (SMO, a transmembrane protein and member of Hedgehog pathway) and phosphodiesterase 4D (PDE4D), respectively [76–79].

### MiRNAs and resistance to platinum drugs

Platinum drugs are a class of cell cycle non-specific drugs, which mainly form platinum-DNA adduction with DNA in cancer cells and result in necrosis or apoptosis of cancer cells. Due to their unique anticancer mechanisms and extensive anticancer spectrum, platinum drugs have been widely utilized in clinic [81, 82]. Currently, platinum-based chemotherapy is a standard treatment for gastric cancer patients. There are a total of three generations of platinum drugs including cisplatin as the first-generation platinum drug, carboplatin as the second-generation platinum drugs, as well as oxaliplatin and loplatin as the third-generation platinum drugs. Multiple miRNAs have been reported to be involved in resistance to platinum drugs in gastric cancer (Table 2).

**Table 2:** MiRNAs and platinum drugs resistance in gastric cancer

MiRNAs	Expression^1^	Genes and Pathways	Drugs	Reference
miR-20a	↑	*EGR2*	cisplatin	[60]
		*CYLD*		[83]
miR-106a	↑	*PTEN/Akt*	cisplatin	[84]
		*RUNX*		[85]
miR-21	↑	*PTEN/Akt*	cisplatin	[86]
miR-25	↑	*FOXO3a*	cisplatin	[87]
miR-141	↑	*KEAP1*	cisplatin	[88]
miR-223	↑	*FBXW7*	cisplatin	[89]
miR-99a, miR-491	↑	*CAPNS1*	cisplatin	[90]
miR-421	↑	*E-cadherin*, *caspase-3*	cisplatin	[91]
miR-135b-5p	↑	*KLF4*	cisplatin	[92]
miR-135b	↑	*MST1*, *MAPK*	cisplatin	[93]
miR-17-5p	↑	*p21*	cisplatin	[94]
miR-193a-3p	↑	*SRSF2*	cisplatin	[28]
miR-4295	↑	*LRIG1*	cisplatin	[95]
miR-3174	↑	*ARHGAP10*	cisplatin	[96]
miR-27a	↑	*P-gp*, *LRP*, *Bcl-2*	oxaliplatin	[97]
miR-135a	↑	*E2F1*, *DAPK2*	oxaliplatin	[98]
miR-27b, miR-508-5p	↓	*ABCB1*, *CCNG1*, *ZNRD1*	cisplatin	[67]
miR-129-5p	↓	*ABCB1*, *ABCC5*, *ABCG1*	cisplatin	[64]
miR-508-5p	↓	*ABCB1*, *ZNRD1*	cisplatin	[65]
miR-129	↓	*P-gp*	cisplatin	[99]
miR-107	↓	*P-gp*, *cyclin D1*, *c-myc*	oxaliplatin	[69]
miR-15b, miR-16	↓	*Bcl-2*	cisplatin	[70]
miR-181b	↓	*Bcl-2*	cisplatin	[71]
miR-497	↓	*Bcl-2*	cisplatin	[72]
miR-200bc/429 cluster	↓	*Bcl-2*, *XIAP*	cisplatin	[73]
miR-34	↓	*Bcl-2*, *Notch*, *HMGA2*	cisplatin	[74]
miR-449a	↓	*Bcl-2*, *CCDN1*	cisplatin	[100]
miR-1271	↓	*IGF1R*, *IRS1*, *mTOR*, *Bcl-2*	cisplatin	[101]
miR-143	↓	*IGF1R*, *Bcl-2*	cisplatin	[102]
miR-503	↓	*IGF1R*, *Bcl-2*	cisplatin	[103]
miR-23b-3p	↓	*ATG12*, *HMGB2*	cisplatin	[104]
miR-181a	↓	*ATG5*	cisplatin	[105]
miR-30	↓	*LC3-II*	cisplatin	[106]
miR-148a-3p	↓	*RAB12*, *mTOR1*, *AKAP1*	cisplatin	[107]
miR-200c	↓	*E-cadherin*	cisplatin	[75]
		*RhoE*		[108]
		*ZEB2*		[109]
miR-101	↓	*ANXA2*	cisplatin	[110]
		*VEGF-C*		[111]
miR-218	↓	*mTOR*	cisplatin	[112]
		*SMO*	oxaliplatin	[78]
miR-185	↓	*ARC*	cisplatin	[76]
miR-34a	↓	*MET*	cisplatin	[113]
miR-26a	↓	*NRAS*, *E2F2*	cisplatin	[114]
miR-149	↓	*FoxM1*	cisplatin	[115]
miR-524-5p	↓	*SOX9*	cisplatin	[116]
miR-362-5p	↓	*SUZ12*	cisplatin	[117]
miR-198	↓	*FGFR1*	cisplatin	[118]
miR-574-3p	↓	*ZEB1*	cisplatin	[119]
miR-876-3p	↓	*TMED3*	cisplatin	[120]
miR-874	↓	*ATG16L1*	cisplatin	[121]
let-7b	↓	*AURKB*	cisplatin	[122]
miR-122	↓	*ERCC1*	cisplatin	[123]
miR-138-5p	↓	*ERCC1*, *ERCC4*	cisplatin	[48]
miR-192-5p	↓	*ERCC3*, *ERCC4*	cisplatin	[124]

^1^miRNAs either up-regulated (↑) or down-regulated (↓) in platinum drugs resistant gastric cancer cells

Note: This table shows 53 miRNAs whose expression levels and potential targets in platinum drugs resistance of gastric cancer.

Several oncogenic miRNAs can promote resistance to platinum drugs in gastric cancer cells, such as miR-20a, miR-106a, miR-21, miR-25, miR-141, miR-223, miR-421, miR-99a, miR-491, and miR-27a. For example, miR-20a could confer DDP resistance by silencing EGR2 and cylindromatosis (CYLD) in gastric cancer cells [60, 83]. Exogenous expression of miR-106a enhanced the resistance to DDP of gastric cancer cells by inhibiting the PTEN/Akt signaling pathway and tumor suppressive RUNX3 [84, 85]. Similarly, miR-21 has also been found to promote DDP resistance via silencing the PTEN/Akt pathway, while contrary results were observed after administration of PI3K inhibitors suppressing Akt pathway [86]. MiR-25, miR-141 and miR-223, which are significantly up-regulated in SGC-7901/DDP resistant gastric cancer cells, could enhance DDP resistance via suppressing expression levels of FOXO3a, kelch-like ECH-associated protein-1(KEAP1) and F-Box and WD repeat domain containing 7 (FBXW7), respectively [87–89]. MiR-99a and miR-491, which are also overexpressed in SGC-7901/DDP and BGC-823/DDP resistant gastric cancer cells, could promote DDP resistance by directly down-regulating their target gene, calpain small subunit 1 (CAPNS1) [90]. MiR-421, a highly expressed miRNA in advanced gastric cancer patients, could also promote gastric cancer metastasis and DDP resistance through inhibiting E-cadherin and caspase-3 expression, *in vivo* and *in vitro* [91]. In addition, oncogenic miR-135b-5p, miR-135b, miR-17-5p, miR-193a-3p, miR-4295 and miR-3174 confer DDP resistance of gastric cancer cells through silencing Krüppel-like factor 4 (KLF4), mammalian ste20-like kinase 1 (MST1), p21, SRSF2, leucine-rich repeats and immunoglobulin-like domains 1 (LRIG1) and ARHGAP10, respectively [28, 92–96]. For oxaliplatin resistance, the oncogenic miR-27a has been found to enhance MDR properties by inducing MDR1/P-gp, lung resistance protein (LRP) and Bcl-2 expression in gastric cancer [97]. Similarly, miR-135a has also been shown to potentiate oxaliplatin resistance of gastric cancer cells by down-regulating expression of E2F transcription factor 1 (E2F1) and death-associated protein kinase 2 (DAPK2) [98].

Conversely, a number of tumor suppressor miRNAs can reverse platinum drugs resistance of gastric cancer. For instance, miR-27b, miR-508-5p, miR-129-5p and miR-129, have been found to reverse gastric cancer resistance to DDP mainly through affecting expression/function of the ABC transporters family members [64, 65, 67, 99]. Similarly, miR-107 has been found to increase the sensitivity of gastric cancer cells to oxaliplatin through inhibiting expression of P-gp, cyclin D1 and c-myc [69]. By inhibiting the Bcl-2 signaling pathway, several tumor suppressor miRNAs, including miR-15b, miR-16, miR-181b, miR-497, the miR-200bc/429 cluster, miR-34 as well as miR-449a, have been found to sensitize gastric cancer cells to DDP [70–74, 100]. Activation of the IGF1R/IRS1 pathway has also been identified to promote drug resistance of multiple malignancies, including gastric cancer. A variety of miRNAs, such as miR-1271, miR-143 and miR-503, have been observed to be involved in the IGF1R/IRS1 pathway-mediated DDP resistance of gastric cancer [101–103]. Recently, emerging evidences indicate that miRNAs are involved in MDR controlling by regulating autophagy through targeting autophagy related genes. In gastric cancer, for example, exogenous overexpression of miR-23b-3p has been found to reverse DDP resistance by modulating expression of autophagy related 12 (ATG12) and high mobility group box 2 (HMGB2) [104]. Similarly, miR-181a, miR-30 and miR-148a-3p have been found to enhance DDP sensitivity of gastric cancer cells through suppressing expression of ATG5, LC3-II, RAB12 and mTOR1, respectively [105–107]. In addition, ectopic miR-200c has been found to sensitize SGC7901/DDP resistance gastric cancer cells to DDP through inhibiting expression of E-cadherin, Rho family GTPase 3 (RhoE) and ZEB2 [75, 108, 109]. By modulating Annexin A2 (ANXA2) and VEGF-C expression, tumor suppressor miR-101 has been found to reverse DDP resistance of gastric cancer cells [110, 111]. As for miR-218, its overexpression has been found to enhance sensitivity to DDP and oxaliplatin by targeting mTOR and SMO, respectively [78, 112]. Moreover, exogenous expression of tumor suppressor miR-185, miR-34a, miR-26a, miR-149 and miR-524-5p in gastric cancer cells can also improve DDP sensitivity. Their potential target genes are ARC, mesenchymal-epithelial transition factor (MET), neuroblastoma RAS viral oncogene homolog (NRAS), E2F transcription factor 2 (E2F2), Forkhead box M1 (FoxM1) and SOX9, respectively [76, 113–116]. Similarly, tumor suppressor miR-362-5p, miR-198, miR-574-3p, miR-876-3p, miR-874 and let-7b have been reported to reverse DDP resistance of gastric cancer cells via silencing suppressor of zeste 12 protein (SUZ12), fibroblast growth factor receptor 1 (FGFR1), zinc finger E-box binding homeobox transcription factor 1 (ZEB1), TMED3, autophagy-related 16-like 1 (ATG16 L1) and AURKB, respectively [117–122]. In addition, via targeting excision repair cross-complementing (ERCC), exogenous over-expression of tumor suppressor miR-122, miR-138-5p and miR-192-5p could also reverse DDP resistance of gastric cancer [48, 123, 124].

### MiRNAs and 5-FU resistance

5-FU is commonly applied for the treatment of gastric cancer in clinic. 5-FU could disturb DNA replication via suppressing thymidylate synthase (TS), thereby leading to apoptosis and cell cycle arrest [125, 126]. It has been found that multiple oncogenic or tumor suppressive miRNAs are involved in 5-FU resistance (Table 3).

**Table 3:** MiRNAs and 5-FU resistance in gastric cancer

MiRNAs	Expression^1^	Genes and Pathways	Reference
miR-BART20-5p	↑	*BAD*	[56]
miR-193-3p	↑	*PTEN*	[127]
miR-147	↑	*PTEN*	[128]
miR-17	↑	*DEDD*	[129]
miR-129-5p	↓	*ABCB1*, *ABCC5*, *ABCG1*	[64]
miR-508-5p	↓	*ABCB1*, *ZNRD1*	[65]
miR-27b, miR-508-5p	↓	*ABCB1*, *CCNG1*, *ZNRD1*	[67]
miR-107	↓	*P-gp*, *cyclin D1*, *c-myc*,	[69]
miR-181b	↓	*Bcl-2*	[71]
miR-429	↓	*Bcl-2*	[130]
miR-200c	↓	*E-cadherin*	[75]
miR-218	↓	*SMO*	[78]
miR-23b-3p	↓	*ATG12*, *HMGB2*	[104]
miR-31	↓	*RhoA*	[131]
		*ZH2*	[132]
		*E2F6*, *SMUG1*	[133]
miR-197	↓	*MAPK1*	[134]
miR-BART15-3p	↓	*TAX1BP1*	[135]
miR-195-5p	↓	*ZNF139*	[136]
miR-204	↓	*TGFBR2*	[137]
miR-623	↓	*CCND1*	[138]
miR-939	↓	*SLC34A2/Raf/MEK/ERK*	[139]
miR-124	↓	*EZH2*	[140]

^1^ miRNAs either up-regulated (↑) or down-regulated (↓) in 5-FU resistant gastric cancer cells

Note: This table shows 21 miRNAs whose expression levels and potential targets in 5-FU resistance of gastric cancer.

Several oncogenic miRNAs promote 5-FU resistance of gastric cancer cells, such as miR-BART20-5p, miR-193a-3p, miR-147 and miR-17. It has been found that EB virus miRNA miR-BART20-5p significantly increased 5-FU resistance of AGS1 gastric cancer cells by inhibiting BAD expression [56]. Similarly, miR-193-3p and miR-147 could promote 5-FU resistance of gastric cancer cells via directly suppressing their target gene PTEN [127, 128]. Oncogenic miR-17 has been found to reduce 5-FU sensitivity of gastric cancer cells through silencing expression of DEDD [129].

On the contrary, several tumor suppressor miRNAs can reverse 5-FU resistance of gastric cancer. Accumulated evidences indicate that elevated expression of MDR-related ABC transporters confer 5-FU resistance. It has, for example, been found that tumor suppressor miR-27b, miR-508-5p, miR-129-5p and miR-107 could inhibit expression of certain family member of ABC transporters and, thus, enhance sensitivity of gastric cancer cells to 5-FU [64, 65, 67, 69]. MiR-181b, which is significantly down-regulated in MDR SGC7901/VCR cells, could sensitize gastric cancer cells to VCR and 5-FU through suppressing expression of Bcl-2 [71]. Similarly, Bcl-2 silencing by miR-429 has also been found to sensitize gastric cancer cells to 5-FU [130]. In addition, over-expression of miR-23b-3p, an autophagy-related modulator, has been found to sensitize gastric cancer cells to 5-FU by inhibiting expression of ATG12 and HMGB2 [104]. MiR-31, a pleomorphic tumor suppressor miRNA, has been shown to impair 5-FU resistance of gastric cancer cells via silencing RhoA, zeste homolog 2 (ZH2), E2F6 and SMUG1 [131–133]. Additionally, exogenous expression of miR-197 and miR-BART15-3p could also strongly promote 5-FU chemosensitivity through down-regulating expression of mitogen-activated protein kinase 1 (MAPK1) and Tax1-binding protein 1 (TAX1BP1), respectively [134, 135]. Tumor suppressor miR-195-5p, miR-204, miR-623, miR-939 and miR-124 have been shown to be involved in overcoming 5-FU resistance of gastric cancer via silencing Zing finger 139 (ZNF139), TGFBR2, cyclin D1 (CCND1), solute carrier family 34 member 2 (SLC34A2) and EZH2, respectively [136–140].

### MiRNAs and VCR resistance

As an anti-microtubule drug, VCR could inhibit tubulin polymerization and disable spindles formation, which leading to mitosis arrest of malignant cells. It has been found that several oncogenic or tumor suppressive miRNAs are involved in VCR resistance (Table 4).

**Table 4:** MiRNAs and vincristine resistance in gastric cancer

MiRNAs	Expression^1^	Genes and Pathways	Reference
miR-19a/b	↑	*PTEN*	[57]
miR-129-5p	↓	*ABCB1*, *ABCC5*, *ABCG1*	[64]
miR-508-5p	↓	*ABCB1*, *ZNRD1*	[65]
miR-27b, miR-508-5p	↓	*CCNG1*, *ABCB1*, *ZNRD1*	[67]
miR-15b, miR-16	↓	*Bcl-2*	[70]
miR-181b	↓	*Bcl-2*	[71]
miR-497	↓	*Bcl-2*	[72]
miR-200bc/429 cluster	↓	*Bcl-2*, *XIAP*	[73]
miR-126	↓	*EZH2*	[77]
miR-647	↓	*ANK2*	[141]
miR-1284	↓	*EIF4A1*	[142]
miR-23b-3p	↓	*ATG12*, *HMGB2*	[104]
miR-101	↓	*ANXA2*	[110]

^1^ miRNAs either up-regulated (↑) or down-regulated (↓) in vincristine resistant gastric cancer cells

Note: This table shows 14 miRNAs whose expression levels and potential targets in vincristine resistance of gastric cancer.

On one hand, oncogenic miR-19a/b has been reported to enhance VCR resistance of gastric cancer cells by suppressing expression of its target gene PTEN [57]. On the other hand, multiple tumor suppressor miRNAs have been found to be able to reverse VCR resistance of gastric cancer. For example, miR-129-5p, miR-508-5p and miR-27b could sensitize resistance gastric cancer cells to VCR via silencing members of ABC transporters [64, 65, 67]. Similarly, tumor suppressor miR-15b, miR-16, miR-181b, miR-497 and the miR-200bc/429 cluster have been found to increase VCR sensitivity through targeting the Bcl-2 family members [70–73]. In addition, ectopic expression of miR-126, miR-647 and miR-1284, which are evidently downregulated in drug-resistant SGC7901/VCR gastric cancer cells, could sensitize gastric cancer cells to VCR by inhibiting expression of EZH2, ANK2 and EIF4A1, respectively [77, 141, 142]. In addition, miR-23b-3p and miR-101 are able to reverse drug resistance of gastric cancer cells to multiple chemotherapeutics including VCR via targeting ATG12/HMGB2 as well as ANXA2 [104, 110].

### MiRNAs and PTX resistance

PTX is a highly effective cytotoxin to tubulin and can “freeze” the mitotic spindle apparatus of rapidly divided cancer cells, which leading to G2/M cell cycle arrest. PTX is one of the first-line chemotherapeutic reagents to treat gastric cancer. A variety of miRNAs have been reported to be involved in PTX resistance of gastric cancer (Table 5).

**Table 5:** MiRNAs and paclitaxel resistance in gastric cancer

MiRNAs	Expression^1^	Genes and Pathways	Reference
miR-21	↑	*P-gp*	[143]
miR-23a	↑	*IRF1*	[144]
miR-590-5p	↑	*RECK*, *AKT*, *ERK*	[145]
miR-155-5p	↑	*GATA3*, *TP53INP1*	[146]
miR-34c-5p	↓	*MAPT*	[147]
miR-34a	↓	*E2F5*	[148]
miR-495	↓	*ABCB1*	[149]

^1^ miRNAs either up-regulated (↑) or down-regulated (↓) in paclitaxel resistant gastric cancer cells

Note: This table shows 7 miRNAs whose expression levels and potential targets in paclitaxel resistance of gastric cancer.

Several oncogenic miRNAs can promote PTX resistance, such as miR-21, miR-23a and miR-155-5p. A significantly elevated miR-21 expression was observed in PTX resistant SGC7901/PTX gastric cancer cells compared to the parent SGC7901 cells. MiR-21 could dramatically inhibit apoptosis induced by PTX, partly through regulating P-gp expression [143]. MiR-23a, which is dramatically up-regulated in human gastric cancer tissues, has been shown to weaken PTX-induced apoptosis and accelerate proliferation of BGC823 and MGC803 gastric cancer cells via suppressing expression of interferon regulator factor 1 (IRF1) [144]. In addition, exogenous expression of miR-590-5p has been found to impair PTX sensitivity of gastric cancer cells by silencing RECK and the AKT/ERK signaling pathway [145]. Recently, miR-155-5p was identified as an enriched miRNA in exosomes deriving from PTX-resistant MGC-803R gastric cancer cells. Exogenous expression of miR-155-5p confers chemoresistance and EMT in PTX-sensitive MGC-803S cells through targeting and suppressing GATA binding protein 3 (GATA3) and tumor protein p53-inducible nuclear protein 1 (TP53INP1) [146].

Meanwhile, multiple tumor suppressor miRNAs have been found to be able to reverse PTX resistance in gastric cancer. For instance, miR-34c-5p, which was found to be significantly down-regulated in PTX-resistant gastric cancer tissues, could sensitize SGC7901/VCR resistant cells to PTX via targeting and suppressing microtubule-associated protein tau (MAPT) [147]. Tumor suppressor miR-34a has also been shown to sensitize gastric cancer cells to PTX via inhibiting expression of oncoprotein E2F5 [148]. Similarly, ectopic expression of tumor suppressor miR-495 has been found to potentiate PTX-ADR sensitivity in MDR SGC7901R gastric cancer cells through modulating expression of ABCB1 [149].

### MiRNAs and resistance to targeted therapy drugs

Target-therapy provides new therapeutic strategies for advanced gastric cancer. Multiple targeted therapy drugs have been developed to treat cancers including gastric cancer, such as cetuximab and panitumumab (anti-EGFR monoclonal antibodies [MoAbs]), trastuzumab and pertuzumab (anti-HER2 MoAbs), lapatinib (anti-HER2 and EGFR tyrosine kinase dual inhibitor), bevacizumab (anti-VEGF-A MoAb) and ramucirumab (anti-VEGFR-2 MoAb). Among these drugs, trastuzumab and ramucirumab have been found to improve prognosis of advanced gastric cancer patients [34, 150–153]. Emerging evidences have demonstrated that several miRNAs have been involved in resistance to targeted therapy drugs in gastric cancer, such as trastuzumab and lapatinib (Table 6).

**Table 6:** MiRNAs and targeted therapy drugs resistance in gastric cancer

MiRNAs	Expression^1^	Genes and Pathways	Drugs	Reference
miR-21	↑	*PTEN*	trastuzumab	[154]
miR-125b	↑		trastuzumab	[155]
miR-223	↑	*FBXW7*	trastuzumab	[156]
miR-200c	↓	*ZEB1*, *ZEB2*	trastuzumab	[157]
miR-16	↓	*CCNJ*, *FUBP1*	trastuzumab, lapatinib	[158]
miR-494	↓	*FGFR2*	lapatinib	[159]

^1^ miRNAs either up-regulated (↑) or down-regulated (↓) in targeted drugs resistant gastric cancer cells

Note: This table shows 6 miRNAs whose expression levels and potential targets in targeted therapy drugs resistance of gastric cancer.

Trastuzumab, a US Food and Drug Administration (FDA) approved anti-human HER2 MoAb, combined with conventional chemotherapy has been broadly used in advanced or metastatic gastric cancer patients with HER2 overexpression/amplification. Through inhibiting PTEN expression, oncogenic miR-21 has been found to confer trastuzumab resistance in HER2-positive gastric cancer cells [154]. MiR-125b, which is markedly up-regulated in gastric cancer tissues, is significantly associated with trastuzumab resistance and poor prognosis in HER2-positive gastric cancer patients [155]. Similarly, oncogenic miR-223 could decrease trastuzumab sensitivity of gastric cancer through suppressing expression of FBXW7 [156]. In addition, exogenous expression of tumor suppressor miR-200c has been found to sensitize gastric cancer cells to trastuzumab by targeting and downregulating ZEB1 and ZEB2 [157]. Similarly, through modulating cyclin J (CCNJ) and far upstream element-binding protein 1 (FUBP1), exogenous expression of miR-16 could reverse the resistance to trastuzumab and lapatinib in HER2 positive gastric cancer cells [158]. For lapatinib resistance, it has been reported that tumor suppressor miR-494 could not only reverses lapatinib resistance but also inhibit formation of cancer-initiating cells (CICs) via down-regulating expression of receptor tyrosine kinase fibroblast growth factor receptor 2 (FGFR2) in HER2-positive, FGFR2 overexpressing and lapatinib resistant YCC1-F gastric cancer cells [159].

### MiRNAs and resistance to other drugs

Etoposide, mitomycin and capecitabine are also commonly used to treat gastric cancer. Several miRNAs are involved in resistance resistance to these drugs (Table 7). It has been found that by targeting and suppressing Bcl-2 expression, tumor suppressor miR-15b, miR-16, miR-181b, miR-497 and the miR-200bc/429 cluster could sensitize gastric cancer cells to etoposide or mitomycin [70–73]. Significantly increased plasma miR-17-92 cluster levels has been found in advanced gastric cancer patients. Whereas, there was obvious decrease of these miRNAs in chemosensitive individuals after oxaliplatin/capecitabine (XELOX) chemotherapy, indicating they might be used as biomarkers of XELOX treatment [160].

**Table 7:** miRNAs and resistance to other drugs in gastric cancer

MiRNAs	Expression^1^	Genes and Pathways	Drugs	Reference
miR-15b, miR-16	↓	*Bcl-2*	etoposide, mitomycin	[70]
miR-181b	↓	*Bcl-2*	etoposide	[71]
miR-497	↓	*Bcl-2*	etoposide	[72]
miR-200bc/429 cluster	↓	*Bcl-2*, *XIAP*	etoposide	[73]
miR-17-92 cluster	↑		oxaliplatin/capecitabine	[160]

^1^ miRNAs either up-regulated (↑) or down-regulated (↓) in other chemo-drugs resistant gastric cancer cells

Note: This table shows 6 miRNAs whose expression levels and potential targets in other chemo-drugs resistance of gastric cancer.

## LncRNAs and chemoresistance

LncRNAs are a class of ncRNAs longer than 200 nt and have no protein coding potential. LncRNAs play a part in regulating various cellular processes. A number of lncRNAs have been identified to be abnormally expressed in gastric cancer and involved in chemoresistance via regulation of different target genes. Several oncogenic lncRNAs, such as prostate cancer-associated transcript 1 (PCAT-1), SNHG5, BCAR4, GHET1, HOTAIR, plasmacytoma variant translocation 1 (PVT1), metastasis-associated lung adenocarcinoma transcript 1 (MALAT1), urothelial carcinoma-associated 1 (UCA1) and nuclear paraspeckle assembly transcript 1 (NEAT1), as well as some tumor suppressor lncRNAs, have been shown to participate in gastric cancer chemoresistance.

### LncRNAs and resistance to platinum drugs

Multiple (Table 8). For instance, lncRNA PCAT-1, a highly expressed lncRNA in DDP-resistant gastric cancer tissues and cells, promotes DDP resistance of gastric cancer cells via epigenetically suppressing PTEN expression by recruiting EZH2, as well as regulating the miR-128/ZEB1 axis [161, 162]. Similarly, an evidently high expression lncRNA DANCR has been identified in SGC7901/DDP and BGC823/DDP DDP-resistant gastric cancer cells. Knockdown of DANCR in these cells promote apoptosis and inhibit cell proliferation. On the contrary, overexpressed DANCR could up-regulated expression of MDR genes MDR1 and MRP1 in DDP-induced SGC901 and BGC823 cells [163]. LncRNA SNHG5 reduced DDP sensitivity of BGC823 and SGC7901 gastric cancer cells through up-regulating expression of MDR1, MRP1 and Bax as well as downregulating Bcl-2 expression [164]. In addition, lncRNAs GHET 1[165], AK022798 [166], antisense non-coding RNA in the INK4 locus (ANRIL) [167], UCA 1[168] and HULC [169] also conferred DDP resistance of gastric cancer cells via modulating expression of multiple drug resistance-related genes. LncRNA HOTAIR, which was significantly up-regulated in DDP-resistant gastric cancer cells and tissues, could regulate chromatin status and contribute to DDP resistance of gastric cancer, through activating PI3K/Akt/MRP1 and Wnt/β-catenin signaling pathways [170, 171]. Interestingly, lncRNA XLOC_006753, which is highly expressed in gastric cancer patients and MDR SGC-7901/DDP gastric cancer cells, could promote resistance to DDP through regulating the PI3K/AKT/mTOR pathway [172]. Onco-lncRNAs zinc finger antisense 1 (ZFAS1) and BCAR4 could promote DDP resistance of gastric cancer via activating the Wnt signaling pathway [173, 174]. LncRNA MALAT1 could function as a sponge RNA of miR-23b-3p and conferred chemoresistance of gastric cancer cells through attenuating the inhibitory effects of miR-23b-3p on expression of *autophagy related 12* (*ATG12*) and promote autophagy of gastric cancer cells [175]. LncRNA *PVT1* is another chemoresistance-related gene and has been found to promote DDP resistance through increasing expression of MDR1, MRP, mTOR and hypoxia inducing factor 1α (HIF-1α) [176–178]. Through regulting the miR-361/ABCB1 axis, lncRNA BLACAT1 has been found to confer oxaliplatin resistance [179].

**Table 8:** LncRNAs and platinum drugs resistance in gastric cancer

LncRNAs	Expression^1^	Genes and Pathways	Drugs	Reference
PCAT-1	↑	*EZH2*, *PTEN*	cisplatin	[161]
		*miR-128/ZEB1*		[162]
DANCR	↑	*MDR1*, *MRP1*	cisplatin	[163]
SNHG5	↑	*Bax*, *MDR1*, *MRP1*, *Bcl-2*	cisplatin	[164]
GHET1	↑	*Bax*, *Bcl-2*, *MDR1*, *MRP1*	cisplatin	[165]
AK022798	↑	*MRP1*, *P-gp*	cisplatin	[166]
ANRIL	↑	*MDR1*, *MRP1*	cisplatin	[167]
UCA1	↑	*miR-27b*	cisplatin	[168]
HULC	↑		cisplatin	[169]
HOTAIR	↑	*miR-126/PI3K/AKT/MRP1*	cisplatin	[170]
		*miR-34a*, *PI3K/Akt*,*Wnt/β-catenin*	cisplatin	[171]
XLOC_006753	↑	*PI3K/AKT/mTOR*	cisplatin	[172]
ZFAS1	↑	*Wnt/β-catenin*	cisplatin	[173]
BCAR4	↑	*Wnt*	cisplatin	[174]
MALAT1	↑	*miR-23b-3p/ATG12*	cisplatin	[175]
PVT-1	↑	*MDR1*, *MRP*, *mTOR*, *HIF-1α*	cisplatin	[176]
BLACAT1	↑	*miR-361/ABCB1*	oxaliplatin	[179]
CASC2	↓	*miR-19a*	cisplatin	[180]
CRAL	↓	*miR-505/CYLD/AKT*	cisplatin	[181]

^1^lncRNAs either up-regulated (↑) or down-regulated (↓) in platinum drugs resistant gastric cancer cells

Note: This table shows 17 lncRNAs whose expression levels and underlying pathways in platinum drugs resistance of gastric cancer.

By contrast, a number of tumor suppressor lncRNAs can reverse platinum drugs resistance of gastric cancer. For instance, significantly diminished expression of lncRNA CASC2 was observed in DDP-resistant gastric cancer tissues and cells. Exogenous expression of lncRNA CASC2 has been found to enhance DDP sensitivity of gastric cancer cells BGC823/DDP and SGC7901/DDP through sponging miR-19a [180]. LncRNA, cisplatin resistance-associated lncRNA (CRAL), has also been identified to be downregulated in DDP-resistant gastric cancer cells. Via sponging endogenous miR-505 to upregulate cylindromatosis (CYLD) expression and subsequently inhibiting AKT activation, ectopic CRAL expression can weaken DDP resistance of gastric cancer cells by potentiating DDP-induced DNA damage and cell apoptosis *in vitro* and in preclinical models [181].

### LncRNAs and ADR resistance

Multiple onco-lncRNAs, such as HOTAIR, CASC9, MRUL, UCA1, D63785, NEAT1 and HULC, are involved in ADR resistance in gastric cancer (Table 9). For example, via inhibiting miR-217 expression, lncRNA HOTAIR can promote ADR resistance of gastric cancer cells [182]. LncRNA *cancer susceptibility candidate 9* (*CASC9*), whose expression is associated with poor differentiation, invasion and lymph node metastases of gastric cancer, has been reported to promote resistance to ADR of gastric cancer cells through up-regulating expression of MDR1 protein [183]. MDR-related and upregulated lncRNA (MRUL), which is significantly up-regulated in MDR SGC7901/ADR and SGC7901/VCR gastric cancer cells, has been found to promote ABCB1 expression and, thus, resistance to ADR and VCR [184]. Interestingly, lncRNA UCA1 has been shown to contribute to ADR resistance through regulating apoptosis-related gene PARP1 and Bcl-2 or via sponging miR-27b [168, 185]. It has been found that lncRNA D63785, functions as a competitive endogenous RNA (ceRNA) of miR-422a, could promote ADR resistance through blocking miR-422-dependent suppression of myocyte enhancer factor-2D (MEF2D) [186]. In addition, lncRNAs HULC and NEAT1 are also involved in enhancing ADR resistance of gastric cancer cells [169, 187]. Recently, lncRNA regulator of reprogramming (ROR) has been found to confer the resistant to ADR and VCR by up-regulating MRP1 expression in gastric cancer cells and correlated to poor patient outcomes [188].

**Table 9:** LncRNAs and adriamycin resistance in gastric cancer

LncRNAs	Expression^1^	Genes and Pathways	Reference
HOTAIR	↑	*miR-217*	[182]
CASC9	↑	*MDR1*	[183]
MRUL	↑	*ABCB1*	[184]
UCA1	↑	*PARP*, *Bcl-2*	[185]
		*miR-27b*	[168]
D63785	↑	*miR-422a/MEF2D*	[186]
HULC	↑		[169]
NEAT1	↑		[187]
ROR	↑	MRP1	[188]

^1^lncRNAs up-regulated (↑) in adriamycin resistant gastric cancer cells

Note: This table shows 8 lncRNAs whose expression levels and underlying pathways in adriamycin resistance of gastric cancer.

### LncRNAs and 5-FU resistance

It has been found that several oncogenic or tumor suppressive lncRNAs are involved in 5-FU resistance (Table 10). Among these onco-lncRNAs, ANRIL was highly expressed in 5-FU resistant gastric cancer cells BGC823/5-FU and gastric cancer tissues [167]. LncRNAs UCA1 and HULC have also been found to promote 5-FU resistance [168, 169]. XLOC_006753 is an up-regulated lncRNA in gastric cancer patients and 5-FU resistant cells SGC-7901/5-FU, which is not only correlated with tumor size, metastasis, TNM stage and worse prognosis in gastric cancer patients, but also contributed to 5-FU resistance by influencing cell cycle G1/S transition, apoptosis, some markers of MDR and EMT expression, as well as the PI3K/AKT/mTOR signaling [172]. Additionally, oncogenic lncRNAs MALAT1 and PVT-1 could contribute to 5-FU resistance of gastric cancer by modulating expression of the miR-23b-3p/ATG12 axis and Bcl-2, respectively [175, 178]. On the contrary, tumor suppressor lncRNAs can reverse 5-FU resistance in gastric cancer. LncRNA LEIGC has, for example, been found to sensitize gastric cancer cells to 5-FU via inhibiting expression of multiple EMT-related genes [189].

**Table 10:** LncRNAs and 5-FU resistance in gastric cancer

LncRNAs	Expression^1^	Genes and Pathways	Reference
ANRIL	↑	*MDR1*, *MRP1*	[167]
UCA1	↑	*miR-27b*	[168]
HULC	↑		[169]
XLOC_006753	↑	*PI3K/AKT/mTOR*	[172]
MALAT1	↑	*miR-23b-3p/ATG12*	[175]
PVT-1	↑	*Bcl-2*	[178]
LEIGC	↓	*snail*, *slug*, *twist*, *ZEB*, *vimentin*	[189]

^1^lncRNAs either up-regulated (↑) or down-regulated (↓) in 5-FU resistant gastric cancer cells

Note: This table shows 7 lncRNAs whose expression levels and underlying pathways in 5-FU resistance of gastric cancer.

### LncRNAs and PTX resistance

Several oncogenic lncRNAs have been found to promote PTX resistance (Table 11). For example, lncRNA ZFAS1 could enhance PTX resistance of SGC7901 gastric cancer cells by altering the expressions of EMT markers (E-cadherin, N-cadherin, and vimentin) and cell cycle related proteins (cyclin D1, cyclin E and cyclin B1), as well as the Wnt/β-catenin signaling [173]. lncRNA MALAT1 has also been found to confer to PTX resistance through targeting miR-23b-3p and ATG12 in gastric cancer cells [175]. In addition, lncRNAs PVT1 [177], HOTAIR [182] and CASC9 [183] also promoted PTX resistance of gastric cancer through modulating expression of various genes.

**Table 11:** LncRNAs and paclitaxel resistance in gastric cancer

LncRNAs	Expression^1^	Genes and Pathways	Reference
ZFAS1	↑	*Wnt/β-catenin*	[173]
MALAT1	↑	*miR-23b-3p/ATG12*	[175]
PVT-1	↑	*MDR1*, *MRP*, *mTOR*	[177]
HOTAIR	↑	*miR-217*	[182]
CASC9	↑	*MDR1*	[183]

^1^lncRNAs up-regulated (↑) in paclitaxel resistant gastric cancer cells

Note: This table shows 5 lncRNAs whose expression levels and underlying pathways in paclitaxel resistance of gastric cancer.

## CircRNAs and chemoresistance in gastric cancer

CircRNAs, a new class of ncRNAs with the special ring structure, are stably expressed in multiple cells and once considered as transcriptional splicing intermediates, by-products or accidental splicing errors. Accumulating evidences indicated that circRNAs play a key regulatory role in cell physiological process and human diseases, including cancer via sponging miRNAs or interacting with various proteins [190–192]. In addition, the annular structure endows circRNAs with inherent stability, which enables them to accumulate in exosomes and exist stably in peripheral body fluids such as plasma and saliva. All these elucidate the potential of circRNAs as markers for diagnosis and therapy of cancers [193–195]. It has been found that circRNAs not only contribute to development and metastasis of malignances, but also are involved in chemoresistance [196–199]. Several oncogenic circRNAs have been found to participate in drug resistance of gastric cancer (Table 12). For instance, circAKT3 (hsa_circ_0000199), which is derived from exons 8, 9, 10 and 11 of the *AKT3* gene and highly expressed in DDP-resistant gastric cancer cells and tissues, plays a crucial role in DDP resistance of gastric cancer. It has been found that through up-regulating PIK3R1 by sponging miR-198, circAKT3 could confer DDP resistance of gastric cancer cells. Moreover, circAKT3 could promote DNA damage repair and suppress apoptosis of gastric cancer cells *in vitro* and *in vivo*. Additionally, increased expression of circAKT3 was an independent risk factor for disease-free survival (DFS) of gastric cancer patients treated with DDP [200]. Similarly, cirRNA hsa_circ_0081143, which is up-regulated in gastric cancer tissues, could also contribute to DDP resistance by regulating the miR-646/CDK6 pathway. Knockdown of hsa_circ_0081143 inhibited tumorigenesis and remarkably sensitized gastric cancer cells to DDP *in vivo*. In addition, elevated expression of hsa_circ_0081143 is significantly associated with lymph node metastases, advanced TNM stage and worse overall survival (OS) of gastric cancer patients [201]. Through sponging miR-182-5p, a novel circRNA circFN1 (hsa_circ_0058147, deriving from FN1 gene exons 10, 11, and 12) has also been found to promote DDP resistance in gastric cancer [202]. In addition, circRNA circ-PVT1 could facilitate paclitaxel (PTX) resistance of gastric cancer cells via up-regulating ZEB1 mediated by miR-124-3p [23]. Circ MTHFD2, an overexpressed circRNA in pemetrexed (MTA) resistant MGC-803/MTA gastric cancer cells, could enhance the drug resistance of MGC-803/MTA cells by binding to miR-124 and increased protein expression of FDZ5 and MDR-1 [203].

**Table 12:** CircRNAs and drug resistance in gastric cancer

CircRNAs	Expression^1^	Genes and Pathways	Related drug	Reference
circAKT3	↑	miR-198/ PIK3R1	cisplatin	[200]
hsa_circ_0081143	↑	miR-646/CDK6	cisplatin	[201]
circFN1	↑	miR-182-5p	cisplatin	[202]
circ-PVT1	↑	miR-124-3p/ZEB1	paclitaxel	[23]
circ MTHFD2	↑	miR-124/FDZ5/MDR-1	pemetrexed	[203]

^1^ circRNAs up-regulated (↑) in chemo-resistant gastric cancer cells.

Note: This table shows 5 circRNAs whose expression levels and underlying pathways in chemoresistance of gastric cancer.

## Conclusions and future perspectives

**Fig. 1:**
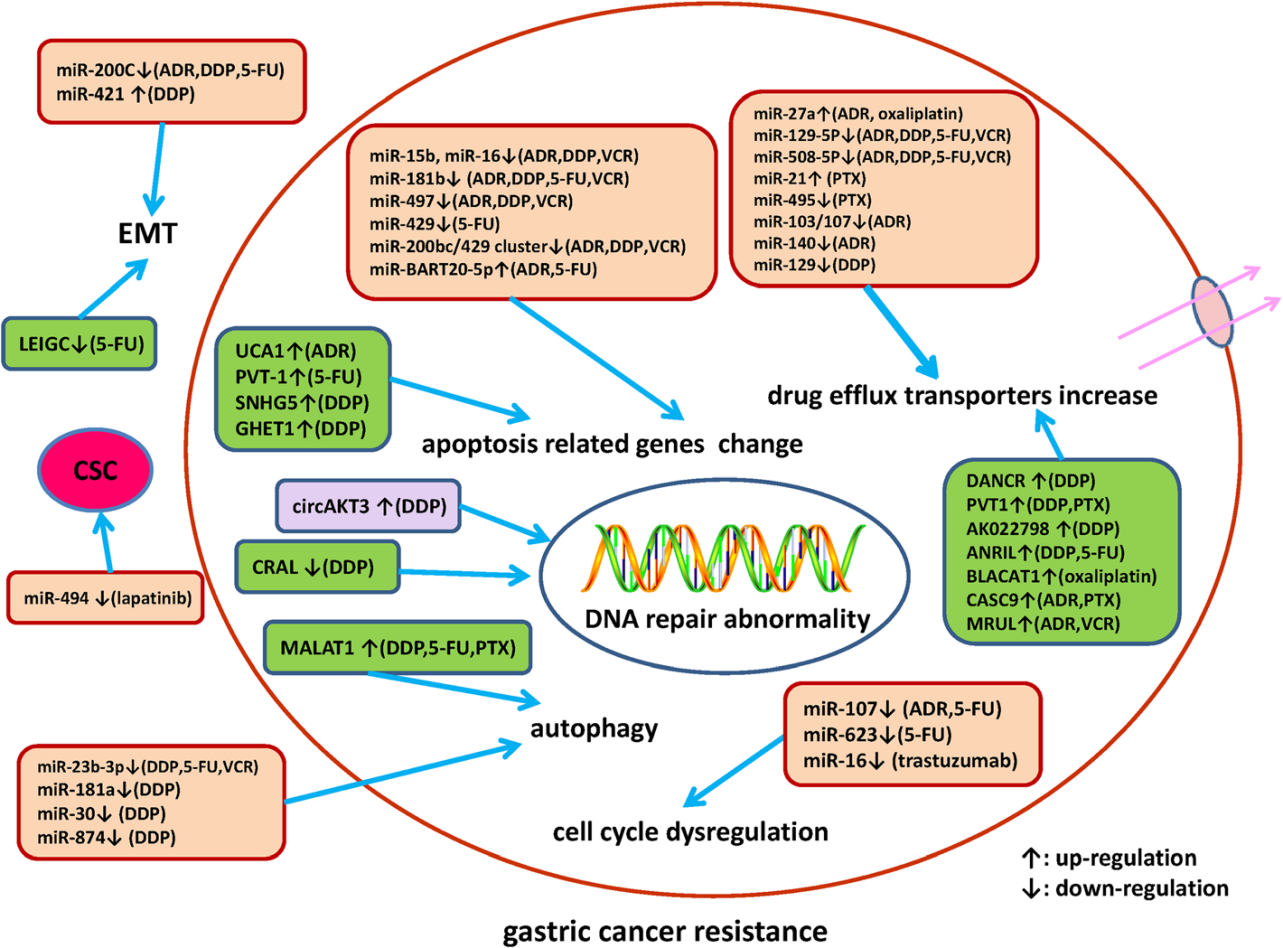
A summary diagram of miRNAs, lncRNAs and circRNAs participated in the drug resistance of gastric cancer. Several miRNAs, lncRNAs and circRNAs have been found to be involved in gastric cancer drug resistance by influencing apoptosis, DNA repair, cell cycle, proliferation, autophagy, epithelial-mesenchymal transition, and cancer stem cell through regulating expression of potential target genes and related signaling pathway

An increasing number of ncRNAs have been identified to be involved in drug resistance of gastric cancer. As shown in Figure 1, the underlying mechanisms of drug resistance-related ncRNAs in gastric cancer are summarized. Treatments targeting these abnormally expressed ncRNAs is a promising approach to reverse drug resistance. Exogenous expression of tumor suppressive ncRNAs or knockdown of oncogenic ncRNAs via small interfering RNAs (siRNAs) or short hairpin RNAs (shRNAs) have been investigated to be applicable in reversing drug resistance of gastric cancer. Combination of ncRNAs-based therapeutic interventions with traditional chemotherapy or targeted therapy may be a promising option to conquer drug resistance in advanced gastric cancer patients. Whereas, it is still a challenge how to choose crucial target ncRNAs from a large amount of candidate ncRNAs. Future translational studies or clinical trials are warrant to develop ncRNAs-based therapeutics, which may eventually improve prognosis of gastric cancer patients via overcoming drug resistance.

## Data Availability

Not applicable.

## Abbreviations

ncRNA: Noncoding RNA
miRNA/miR: MicroRNA
lncRNA: Long noncoding RNA
circRNA: Circular RNA
snRNA: Small nuclear RNA
snoRNA: Small nucleolar RNA
MDR: Multi-drug resistance
CSC: Cancer stem cells
EMT: Epithelial-mesenchymal transition
ADR: Adriamycin
DDP: Cisplatin
5-FU: 5-fluorouracil
VCR: Vincristine
PTX: Paclitaxel
nt: Nucleotide
piRNA: PIWI-interacting RNA
AP-2α: Activating protein 2 alpha
3’-UTR: 3’-untranslated region
mRNA: Messenger RNA
PTEN: Phosphatase and tensin homologue
BLID: BH3-like motif-containing protein, cell death inducer
EGR2: Early growth response 2
TIMP3: Tissue inhibitor of matrix metalloproteinases 3
FADD: Fas-associated death domain
HDAC: Histone deacetylase
ABCB1: ATP-binding cassette, subfamily B, member 1
ZNRD1: Zinc ribbon domain-containing 1
cav-1: Caveolin-1
HMGA2: High-mobility group AT-hook 2
EZH2: Enhancer of zeste homolog 2
PDE4D: Phosphodiesterase 4D
SMO: Smoothened
CYLD: Cylindromatosis
KEAP1: Kelch-like ECH-associated protein-1
FBXW7: F-Box and WD repeat domain containing 7
CAPNS1: Calpain small subunit 1
KLF4: Krüppel-like factor 4
MST1: Mammalian ste20-like kinase 1
LRIG1: Leucine-rich repeats and immunoglobulin-like domains 1
DAPK2: Death-associated protein kinase 2
ATG12: Autophagy related 12
HMGB2: High mobility group box 2
ANXA2: Annexin A2
SUZ12: Suppressor of zeste 12 protein
FGFR1: Fibroblast growth factor receptor 1
ZEB1: Zinc finger E-box binding homeobox transcription factor 1
ERCC: Excision repair cross-complementing
TS: Thymidylate synthase
ZH2: Zeste homolog 2
MAPK1: Mitogen-activated protein kinase 1
TAX1BP1: Tax1-binding protein 1
ZNF139: Zing finger 139
CCND1: Cyclin D1
SLC34A2: Solute carrier family 34 member 2
MAPT: Microtubule-associated protein tau
FDA: US Food and Drug Administration
CCNJ: Cyclin J
FUBP1: Far upstream element-binding protein 1
FGFR2: Fibroblast growth factor receptor 2
PCAT-1: Prostate cancer-associated transcript 1
HOTAIR: HOX transcript antisense gene RNA
MALAT1: Metastasis-associated lung adenocarcinoma transcript 1
UCA1: Urothelial carcinoma-associated 1
NEAT1: Nuclear paraspeckle assembly transcript 1
HIF-1α: Hypoxia inducing factor 1α
CIC: Cancer initiation cell
MRUL: MDR-related and upregulated lncRNA
CASC9: Cancer susceptibility candidate 9
ceRNA: Competitive endogenous RNA
MEF2D: Myocyte enhancer factor-2D
DFS: Disease-free survival
OS: Overall survival
siRNA: Small interfering RNA
shRNA: Short hairpin RNA
